# A Case of Malarial Manifestations Due to Traumatism

**Published:** 1887-08

**Authors:** R. R. Harden

**Affiliations:** Harmony Grove, Ga.


### A CASE OF MALARIAL MANIFESTATIONS DUE TO TRAUMATISM.

BY R. R. HARDEN, M. D., HARMONY GROVE, GA.

Some weeks past, I noticed in a letter from Dr. George M. Sternberg to Dr. Henry C. Coe, in the *New York Medical Journal*. He says: "I think it very probable that the shock of an injury or operation might cause the sudden development of malarial symptoms in one who was under the influence of the malarial poison, or who had been subject to repeated attacks of intermittent fevers."

The case in point is a peculiar one and is this: On the night of July 14th, was called to see Mrs. D., æt. 15. She was married that morning. They retired about 9 o'clock at night. At 11 o'clock of same night I was summoned to see the bride. Arrived in about two-hours, found her exceedingly nervous, thermometer indicating 102 3-5, gave little bromide pot. and tr. valerian; returned next morning about 10 o'clock, temperature and pulse a little higher, but nervous symptoms somewhat abated. Continued treatment. Returned on the morning of the 16th; temperature 103½, pulse 115; learned there had been a considerable remission about middle of last afternoon. Continued treatment.

I returned each morning, and found the pulse and temperature getting higher and higher. On the morning of the 19th the temperature was 105, pulse 120. In the meantime had kept her on the nervous sedatives heretofore mentioned. Bearing Dr.

Sternberg's article in mind, I no longer hesitated. I took the bridegroom out and, as delicate a point as it was, questioned him rigidly. He told me that shortly after retiring he had attempted intercourse, and persisted in the attempt, notwithstanding her importunate cries, until a severe hemorrhage was set up.

Within the last four years I have treated eight cases of remittent and one of intermittent fever in this same house, it being on the west side of a stagnant mill-pond, and receiving the foul east winds from the pond.

I added quinine to the treatment, in free doses, and the symptoms rapidly subsided. Was not this a case of latent malaria? Did not the ineffectual attempt at intercourse, contusing the parts, perhaps rupturing an imperforate hymen (no digital examination was made), produce a condition of traumatism, causing the sudden development of malaria?

I think this an idea for our surgeons, especially those in the more southern latitude, in malarious sections, well enough to consider.

